# Insulin-Induced Severe Lipohypertrophy

**DOI:** 10.1016/j.aace.2024.03.005

**Published:** 2024-03-19

**Authors:** Samir S.E. Ahmed, Mona Vahidi Rad, Joseph Arguinchona, Bithika Thompson

**Affiliations:** 1 Division of Endocrinology and Metabolism, Mayo Clinic Arizona, Scottsdale, Arizona; 2 Division of Internal Medicine, Mayo Clinic Arizona, Phoenix, Arizona

### Case Presentation

A 75-year-old lady with history of type 2 diabetes mellitus for more than 10 years complicated by nephropathy and peripheral neuropathy presented to the endocrine clinic for diabetes care.

Her outpatient diabetic regimen included aspart, 30 units every morning, glargine, 50 units every morning, dapagliflozin 10 mg daily, and dulaglutide 0.75 mg weekly. On presentation A1c was noted to be 8.1% (4.2%-5.6%). On examination, she was noted to have 2 painless, indurated, well demarcated, and easily palpable areas of hyperpigmentation bilaterally surrounding her umbilicus, the left lesion is covered with shiny skin, larger in size and measuring 5 × 5 cm (Fig. A, labeled as 1 and 2).

**Fig:**
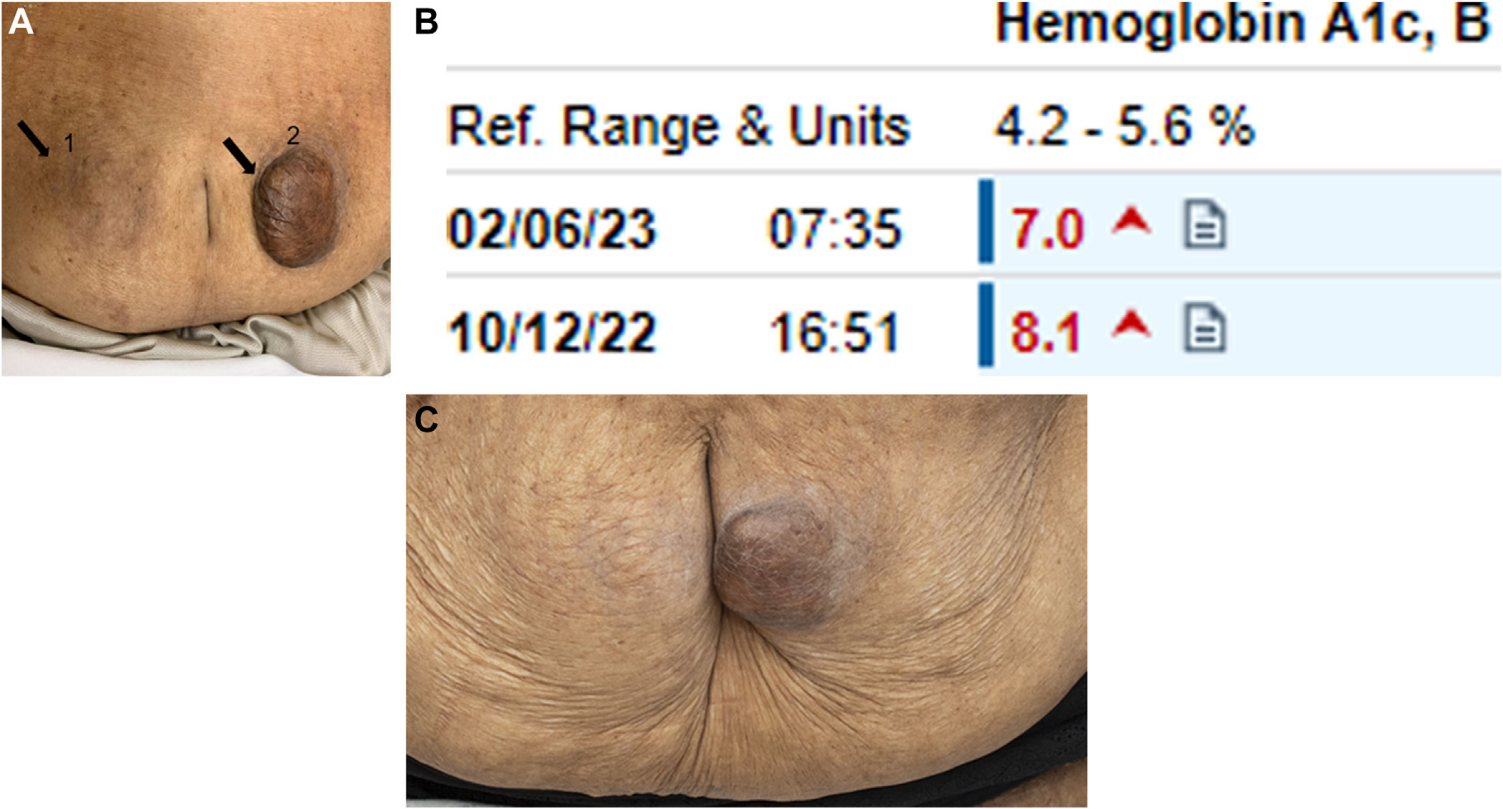
*A*, Photograph showing bilateral periumbilical severe lipohypertrophy more prominent over the left side (2) but also the right side (1) is significant and easily palpable. *B*, Freestyle Libre data showing time in range of 48% with 52% high readings on the initial visit. *C*, Photograph showing Improvement of the bilateral periumbilical lipohypertrophy. In *A* (2) the dark nodule is 2 times the vertical height of the umbilicus and this same nodule in *C* is about one-half the vertical length of the umbilicus.

### What Is the Diagnosis?

#### Answer

Given the history and physical exam findings, the lesions were felt to be secondary to lipohypertrophy, she did endorse only injecting insulin at these 2 sites for many years and mainly over the left-side as she is right-handed. Following the appointment, insulin aspart was adjusted to 20 units with meals plus moderate correction scale. Additionally, dulaglutide was discontinued and tirzepatide was initiated. The patient was provided education on avoiding the affected sites when injecting insulin as well as proper site rotation. Subsequently, over the next couple months her insulin requirement decreased to glargine, 25 units every morning with only sliding scale aspart. She remained on Farxiga, 10 mg daily and tirzepatide. At 6-month follow-up, A1c had decreased to 7.0% (Fig. B). On physical exam, the areas of lipohypertrophy had decreased significantly in size (Fig. C). Lipohypertrophy has been associated with increased risk of hypoglycemia, glycemic variability, and decreased insulin absorption in areas of lipohypertrophy.^1^ This case emphasizes the importance of a routine physical examination, particularly abdominal inspection, when evaluating patients with diabetes on insulin as well as patient education on proper insulin administration and rotation of sites.^2^ Patient education should include avoidance of lipohypertrophy sites, proper site rotation, use of a new needle with each injection, and routine self-inspection of injection sites.^3^

## Disclosure

The authors have no conflicts of interest to disclose.
